# Infective Endocarditis Revealed by Ischemic Stroke in a Young Patient

**DOI:** 10.7759/cureus.96935

**Published:** 2025-11-15

**Authors:** Ismail Bouzekraoui, Myriam Lahraoui, Abdelkader Benhlima, Mouhssine Doumiri, Mourad Amor

**Affiliations:** 1 Neurocritical Care, Ibn Sina University Hospital, Mohammed V University, Rabat, MAR; 2 Cardiology B, Ibn Sina University Hospital, Mohammed V University, Rabat, MAR; 3 Neurocritical care, Ibn Sina University Hospital, Mohammed V University, Rabat, MAR

**Keywords:** hemodynamic instability (hdi), infective endocarditis, : ischemic stroke, neurology and critical care, young adult male

## Abstract

Ischemic stroke is commonly caused by emboli of cardiac or atherosclerotic origin. In young adults, embolic cardiopathies, especially infective endocarditis, are a significant though rare cause. Infective endocarditis represents a contraindication to thrombolysis and therapeutic anticoagulation due to the high risk of hemorrhagic transformation. We report the case of a 26-year-old man who presented an ischemic stroke in the right middle cerebral artery territory due to an infective endocarditis.

## Introduction

Ischemic strokes represent 90% of all cerebrovascular events and represent the main cause of acquired adult disability, followed by dementia, and death worldwide [1,2]. Among the etiological mechanisms, large artery atherosclerosis, cardioembolic events, and small vessel disease dominate [3,4].

Infective endocarditis is a rare and severe cause of ischemic stroke, especially in young individuals with no history of cardiovascular disease. Neurological complications can happen in 20-60% of patients and can be the main presentation in 10-20% [5,6]. These complications carry a high risk of morbidity and mortality, necessitating early diagnosis and management. We report the case of a young man who presented for ischemic stroke caused by infective endocarditis.

## Case presentation

A 26-year-old male patient, with a history of recurrent untreated childhood tonsillitis, presented with a sudden-onset left hemiparesis, facial asymmetry, and dysarthria six hours before his admission to the hospital. Upon admission to the emergency department, he was conscious (GCS 15). The motor assessment found left-sided weakness graded 0/5 in the upper limb and 2/5 in the lower limb, the National Institutes of Health Stroke Scale (NIHSS) score was at 10, and the modified Rankin Scale (mRS) at 4. Blood pressure was 150/70 mmHg, heart rate 90 bpm, oxygen saturation was 97% in ambient air, and blood glucose was 1.35 g/l. Cardiovascular auscultation revealed a systolic murmur at the mitral focus.

**Figure 1:**
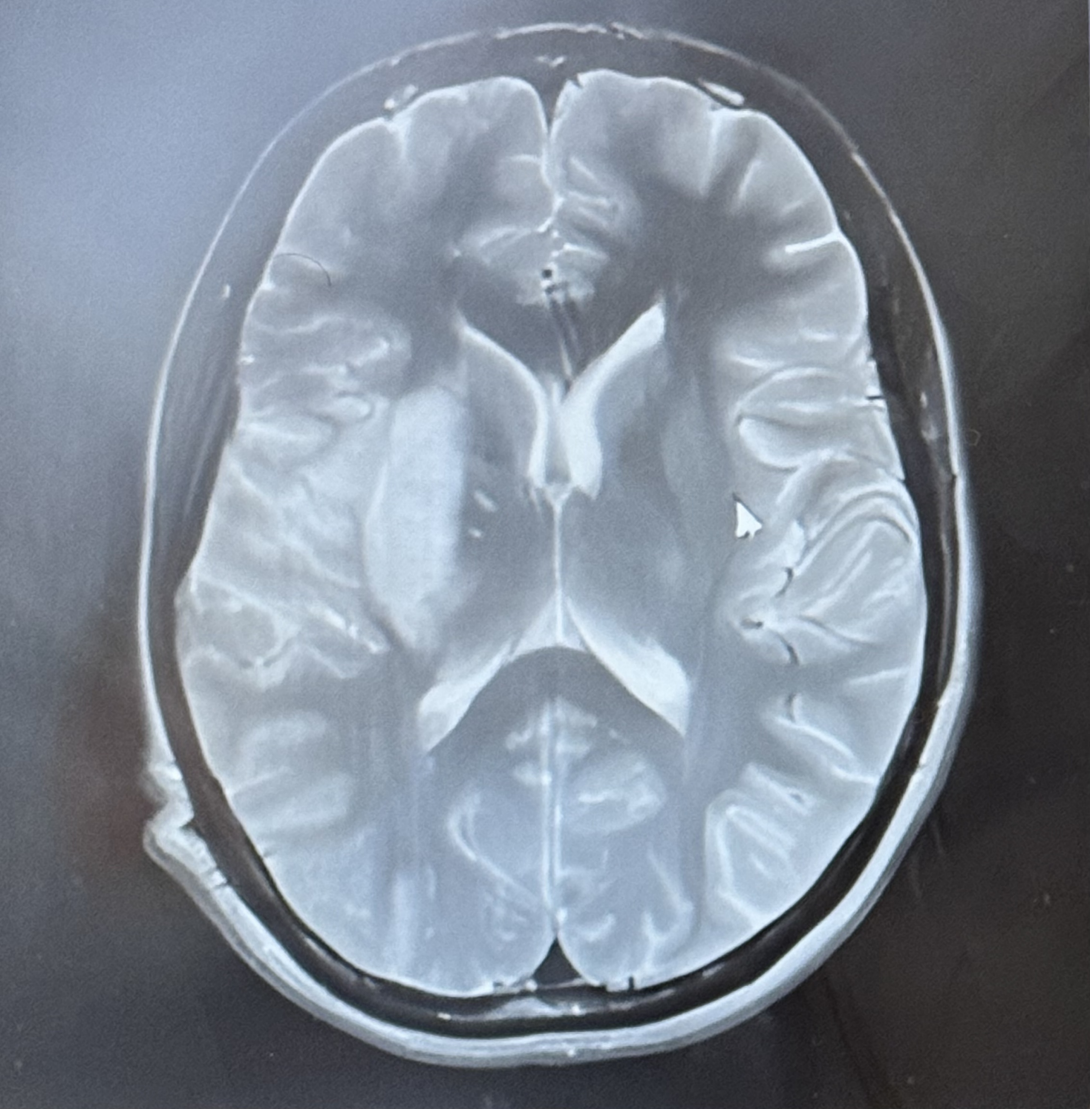
Brain MRI showing an ischemic stroke in the deep territory of the right middle cerebral artery

Brain CT and cerebral MRI angiography (Figure 1) showed an infarction of the deep territory of the right middle cerebral artery without perfusion mismatch (Diffusion+, FLAIR+), contraindicating thrombolysis or mechanical thrombectomy. He was admitted to the neurology department.

**Figure 2:**
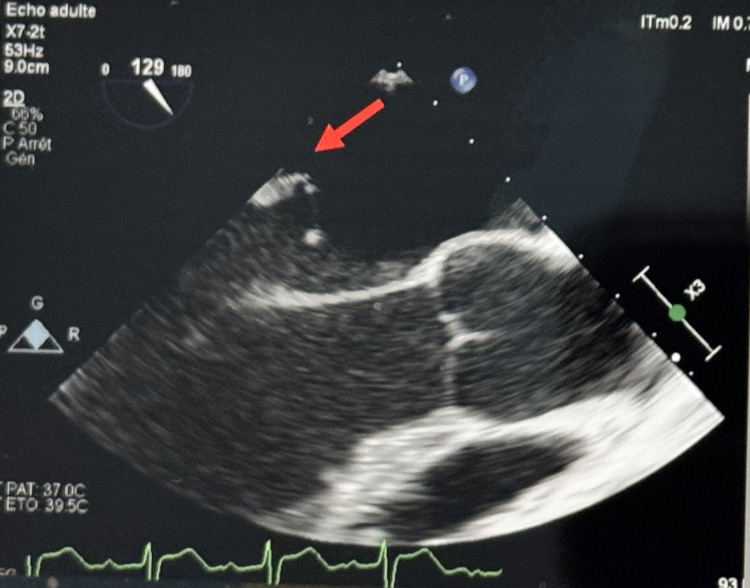
Transoesophageal echocardiography 3 chamber view showing a mobile, filiform structure measuring 17 mm attached to the atrial side of the P2 scallop of the mitral valve

**Figure 3:**
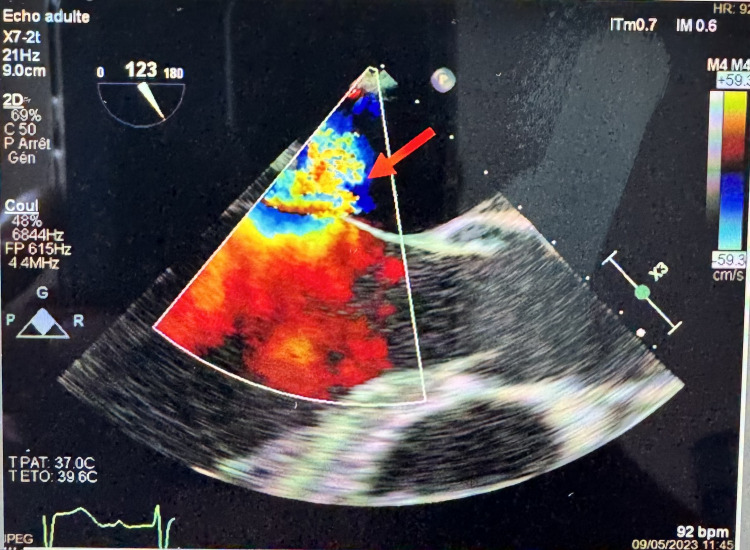
Transoesophageal echocardiography 3 chamber view demonstrating the severity of the mitral regurgitation

On Day 1, he developed hypotension (BP 80/60 mmHg), not responding to fluid resuscitation, requiring ICU transfer. Upon admission to the intensive care unit, we started norepinephrine for a systolic blood pressure target of 140 mmHg. EKG showed sinus rhythm and no conduction disturbance; Doppler ultrasound of the vascular axes of the neck does not reveal any carotid stenosis. Transthoracic echocardiography revealed mitral regurgitation with suspicion of mobile structures on the posterior leaflet. Transesophageal echocardiography confirmed a prolapsed, shredded P2 segment of the mitral valve with mobile vegetations (largest 17 mm) and a patent foramen ovale (Figures 2-3).

Based on the modified Duke criteria (one major: vegetations; three minor: fever >38°C, cardiac predisposition, vascular embolism), the diagnosis of infective endocarditis was established. Empiric antibiotic therapy with amoxicillin at the rate of 2g per 8h (100mg/kg/day) and gentamicin 180mg per day (3mg/kg/day) was initiated after doing blood cultures.

Given the hemodynamic instability and persistent mitral insufficiency, the patient underwent early mitral valve replacement surgery on Day 10 post-stroke. Postoperative evolution was favorable.

## Discussion

Neurological complications are frequent in IE. Ischemic stroke may precede diagnosis, particularly in younger patients. MRI is preferred over CT due to its higher sensitivity [7]. In left-sided IE, silent cerebral infarcts occur in 30% of cases, and neurological complications in up to 65% [7].

Intravenous thrombolysis is contraindicated because of the high risk of hemorrhagic transformation [8,9]. A 2019 meta-analysis found no hemorrhagic events after thrombectomy, compared with 61% with IV thrombolysis and 45% with combined therapy [10]. However, recanalization rates are lower in IE-related stroke (74%) than in other etiologies (87.5%) [11].

The efficacy of antithrombotic therapy remains a subject of debate. Antiplatelet agents may reduce embolic events and are usually continued, while anticoagulation increases hemorrhagic risk, especially after stroke [12,13]. 

Surgery is indicated in severe valvular regurgitation with heart failure, uncontrolled infection, or fungal IE [14]. Early surgery does not increase perioperative stroke risk [11,12] and significantly reduces embolic events (0% vs. 21% with medical therapy) [14].

## Conclusions

Ischemic stroke may be the initial manifestation of infective endocarditis, particularly in younger patients. Optimal management requires a multidisciplinary approach, including comprehensive cardiac evaluation and prompt neuroimaging. In carefully selected cases, early surgical intervention improves prognosis and significantly reduces the risk of recurrence.
